# Study on the chemical compositions and microbial communities of cigar tobacco leaves fermented with exogenous additive

**DOI:** 10.1038/s41598-022-23419-y

**Published:** 2022-11-10

**Authors:** Wanrong Hu, Wen Cai, Zhaojun Zheng, Yuanfa Liu, Cheng Luo, Fang Xue, Dongliang Li

**Affiliations:** 1 Key Laboratory of Chinese Cigar Fermentation, Center of Technology Innovation for Cigar, China Tobacco Sichuan Industrial Co., Ltd, Chengdu, 610000 China; 2 School of Food Science and Technology, Jiangnan University, Wuxi, 214112 China

**Keywords:** Biological techniques, Biotechnology, Microbiology

## Abstract

Fermentation process plays an important role in the biochemical properties and quality of cigar tobacco leaves (CTLs). In industry, exogenous additive (EA) was usually adopted for improving the quality of CTLs during fermentation. However, the mechanism of enhanced quality of CTLs fermented with EA was confused. Herein, the chemical compositions and microbial communities of CTLs during fermentation with EA were analyzed. The increased contents of total nitrogen and total sugar, as well as the improved consumption rate of reducing sugar in CTLs were found with the addition of EA. Besides, fermentation with EA reduced the content of total nonvolatile organic acid, especially unsaturated fatty acid. The contents of total and several representative aroma components were improved. Additionally, the increased abundance of *Staphylococcus* and decreased abundance of *Aspergillus* were detected. Combined with the changes of chemical compositions and microbial communities, it was confirmed that the carbohydrates and alcohols originated from EA promote the enrichment of *Staphylococcus* and accelerate biochemical reactions, such as Maillard reaction and esterification reaction, thus improving the contents and quality of aroma components in CTLs. This study demonstrated the mechanism of enhanced quality of CTLs fermented by EA, which provides more ideas for developing novel and efficient EAs.

## Introduction

Fermentation is widely adopted to adjust the physical and chemical properties of target products based on microbial activity, which has been extensively applied in food processing, medicine and energy production, environmental remediation, and so on^1–3^. Microorganisms in the fermentation system could utilized substrates, such as protein and carbohydrate, and generate various products. Traditional fermentation is a natural process without manual intervention, which is significantly influenced by the environment. Owing to the rapid technological and industrial development, different approaches were used to control the process, including the regulation of fermentation parameters and introduction of starter cultures^4,5^. As a result, the fermentation is more likely proceed with a desired direction, and the consistent quality of products could be obtained.

Tobacco is considered as one of the important industrial and economic crops^6^. Among the various cigarette products, cigar has attracted extensive attention owing to the characteristics of hand-made and particularity of raw material^7^. Cigar was totally made by cigar tobacco leaves (CTLs) without filter and wrapping paper^8^. Thus, the quality of CTLs determines the sensory of cigars. Generally, the production process of cigar includes cultivation, air-curing, fermentation, and rolling. Besides of variety optimization, fermentation is the most effective approach to improve tobacco quality.

According to recent researches, condition optimization has received noticeable attention in the field of CTL fermentation^9,10^. Several important parameters for CTL fermentation, such as temperature, humidity, time, and initial moisture content of tobacco leaves, were studied systematically^9^. However, effectiveness of enhancing the quality of CTLs by traditional fermentation is limited. Based on this trend, adding exogenous additive (EA) to facilitate the fermentation process was proposed. At present, EA for CTLs fermentation includes plant extracts, bacterium, and enzyme preparation, as well as the mixture of above materials^11^. Additives of green tea infusion, diluted milk and rice wine, or *Bacillus cereus* were reported to improve the quality of CTLs, especially the flavor components^12^. Theoretically, EA could affect the growth and metabolism of microorganism community, and alter the biochemical reaction pathways, thus improve the quality of cigar^13^. In fact, mechanism of EA on enhancing the cigar quality has been explored by few researches, and most of them only focused on the effectiveness. Fermentation is complex and enzymatic action of microorganisms as well as chemical interaction would affect the quality of cigar.

Previous studies indicated that several chemical indexes, such as nitrogen, sugar, organic acids, and aroma components, affected the quality of cigar tobacco directly^14–16^. The contents of nitrogen, sugar and organic acids are related to the sweetness and mellowness of CTLs. Besides, different aroma components endow cigars with different flavor profiles. Apart from chemical compositions, microbial communities play an important role in improving the quality of CTLs. The changes of microbial community may demonstrate the mechanism responsible for tobacco quality. Liu et al. found that the bacterial and fungal community structure significantly changed in the CTLs fermentation, and the chemical compositions were influenced by microbial activities^17^. Therefore, it is necessary to analyze the change law of chemical compositions and microbial communities of CTLs with EA during the fermentation process.

Considering the research gap about mechanism of EA on enhancing the cigar quality, our study showed for the first time the effects of EA on the chemical and microbial properties of CTLs. To the best of our knowledge, no studies have systematically compared the changes on sensory qualities, chemical compositions, and microbial communities of CTLs under two different systems of adding water and EA. Therefore, the EA, which was adopted for handmade cigars in actual production at present, was adopted for CTLs fermentation. The effects of EA on the contents of major conventional chemical components (including total nitrogen, nicotine, total sugar, reducing sugar, K, and Cl), nonvolatile organic acids (NOCs), and aroma components in CTLs were investigated. Furthermore, the relevant analysis about microbial communities was conducted. This study aims to provide a systematic investigation about influencing reasons of EA and fermentation process on cigar quality, and propose a theoretical basis for rational development of new EAs and optimization of fermentation process.

## Materials and methods

### Sample collection and tobacco fermentation

A representative kind of CTL sample was used in this study, which was planted in De-yang (Sichuan, China) and named as DX-4. The CTL raw material was obtained after air-curing and possessed the humidity of 17 ~ 20%. The formulated EA for fermentation, which is adopted for handmade cigar products in the Great Wall Cigar Factory (Chengdu, China), was used for this research. Rice wine, fritillaria cirrhosa, and loquat wine were the main components of EA. The reagents used in this study were purchased from Chengdu Kelong Reagent Company and of analytical grade unless stated otherwise.

In a typic fermentation procedure, 217.5 g of EA and 282.5 g of de-ionized water were mixed, which was sprayed evenly on the surface of 5000 g of CTLs. The water content of CTLs was 30 ± 2%. Then, the CTLs were transferred to a linen bag and placed in a constant temperature and humidity incubator (Binder, KBF720). The fermentation was performed under the condition of 35 °C and 75% of relative humidity for 35 days, which was set according to the parameters actually used in the Great Wall Cigar Factory. After 0, 7, 14, 21, 28 and 35 days, 800 g of CTLs was withdraw at given times and denoted as CTL_EA_x (x = 0, 7, 14, 21, 28, and 35, denoted the fermented time). For comparison, control samples without EA replaced by de-ionized water were obtained by the same procedure, which were named as CTL_W_x (x = 0, 7, 14, 21, 28, and 35, denoted the fermented time). All samples were stored in a refrigerator at − 80 °C for further use.

### Sensory quality evaluation

Sensory quality evaluation of CTL samples was performed by a six members-trained panel. Nine indicators including aroma quality, aroma concentration, smoke concentration, strength, cleanliness, offensive odor, aftertaste, ash color, and combustibility were assessed. Sensory characteristic scales were calculated by using a 9-point hedonic scale, and a higher score represented the better performance of the corresponding index. The scores were discussed and agreed by all panelists.

### Determination of chemical components

Six conventional chemical components in tobacco, including the total nitrogen, nicotine, total sugar, reducing sugar, K, and Cl, were usually of general concern in the tobacco industry. Thus, contents of the above-mentioned chemical components were analyzed in this study firstly. A continuous flow analytical system according to the Tobacco Industry Standard (YC/T161-2002, YC/T468-2013, YC/T159-2019, YC/T 217-2007, and YC/T 162-2011) was adopted^9^.

The composition and content of nonvolatile organic acid (NOC) were determined by gas chromatograph (GC) equipped with hydrogen flame ionization detector (FID), since the CTL sample was treated with sulfuric acid–methanol method in advance^18^. A column DB-5MS (60 m × 0.25 mm I.D. × 0.25 µm film thickness) was used. Helium was used as a carrier gas and the flow rate was 1.5 mL min^−1^. The temperature of injection port was 280 °C. The temperature program of column was 40 °C (3 min), 40–280 °C (10 °C min^−1^), and 280 °C (30 min).

### Determination of aroma components

Gas Chromatography-Mass Spectrometry (GC–MS) was used to determine the contents of aroma components in CTLs. Acetonitrile extraction and internal standard method were adopted. In detail, the CTLs were ground into powder firstly. 2 g of the CTL powder sample was mixed with 10 mL of de-ionized water in a 50 mL centrifuge tube for 10 min. Then, 10 mL of acetonitrile (chromatographical grade) and 50 μL of internal standard working solution (phenethyl acetate: 9.06 mg mL^−1^) was pipetted into the centrifuge tube, which was shaken at 2000 rpm for 120 min in a vortex oscillator and cooled at 4 °C for 10 min subsequently. Afterwards, 4 g of anhydrous magnesium sulfate, 1 g of sodium chloride, 1 g of sodium citrate, and 0.5 g of disodium hydrogen citrate were added into the cooled cube, which was shaken using a vortex oscillator immediately to prevent anhydrous magnesium sulfate from agglomerating. Then, 1 mL of extract supernatant was pipetted from the centrifuge cube and mixed with 150 mg of anhydrous magnesium sulfate. Finally, the supernatant from magnesium sulfate suspension was collected for GC–MS analysis.

The GC–MS system (Agilent, 7890B-5977) was coupled with an Agilent DB-5MS column (60 m × 0.25 mm, 1.00 μm). Chromatographic conditions: the temperature of injection port was 290 °C, the temperature program of column was 60–250 °C (2 °C min^−1^), 250–290 °C (5 °C min^−1^), and 290 °C (20 min). The interface was kept at 290 °C. Qualitative analysis was performed in the electron-impact (EI) mode at 70 eV potential using 26–400 amu for qualitative analysis.

### DNA extraction and sequencing

The DNA extraction steps were performed based on previous reports^16,19,20^. Total microbial genomic DNA was extracted from microorganism in CTL samples according to the manufacturer's instruction of E.Z.N.A.® Soil DNA Kit (Omega Bio-Tek, Norcross, GA, U.S.). The integrity of extracted DNA was checked by 1% agarose gel electrophoresis, while the concentration and purity of DNA were determined by NanoDrop 2000 UV–vis spectrophotometer (Thermo Scientific, Waltham, MA, USA). The V3-V4 regions of the bacterial 16S rRNA gene were amplified using the primers 338F (5ʹ- ACTCCTACGGGAGGCAGCAGCAGG -3ʹ) and 806R (5ʹ-GACTACHVGGGTWTCTAAT-3ʹ). The fungal internal transcribed spacer gene was amplified using the primers ITS1F (5ʹ-CTTGGTCATTTAGAGGAAGTAA-3ʹ) and ITS2R (5ʹ-GCTGCGTTCTTCATCGATGC-3ʹ). Then, the PCR products were analyzed using 2% agarose gel electrophoresis and recovered using an AxyPrep DNA Gel Extraction Kit (Axygen Biosciences, Union City, CA, USA). Finally, the purified amplicons were sequenced using the Illumina MiSeq platform.

### Bioinformatics analysis

Fastp and FLASH software were used for checking and merging the raw gene sequencing reads. The sequences were identified as the same operational taxonomic units (OTUs) with 97% similarity threshold using Usearch (version 7.0 http://drive5.com/uparse/). Besides, each sequence was classified and noted using the RDP Classifier (http://rdp.cme.msu.edu/) and Silva database with a confidence threshold of 70%.

### Statement

Permissions for plant material collection was obtained. The collection of plant material complies with relevant institutional, national, and international guidelines and legislation.

## Results and discussion

### Sensory quality evaluation results

As shown in Table 1, the tobacco raw material used in this experiment exhibited medium concentration and strength of smoke, as well as a relatively low aroma quality and concentration. Therefore, it is necessary to improve the quality of tobacco leaves by fermentation with EA. It can be seen that the addition of EA could improve the sensory quality of tobacco leaves, since the sensory scores of EA-group samples were higher than those in water-group. Notably, aroma quality, cleanliness, and aftertaste were enhanced with the addition of EA. Besides, with the extension of fermentation time, the sensory assessment score of CTLs increased first and then decreased, and reached the highest level in 21 days, indicating that excessive fermentation was not conducive to the improvement of sensory quality of cigar.

**Table 1:** Sensory evaluation results of CTL samples.

Samples	Score (0–9)									Total score
	Aroma quality	Aroma concentration	Smoke concentration	Strength	Cleanliness	Offensive odor	Aftertaste	Ash color	Combustibility	
CTL_W_0	5.5	5.9	6.0	6.0	5.2	5.5	6.2	7.0	7.0	54.3
CTL_W_7	6.4	6.5	6.3	6.0	5.7	5.6	6.4	7.0	7.0	56.9
CTL_W_14	7.2	7.1	6.4	5.8	6.2	5.9	6.7	7.0	7.0	59.3
CTL_W_21	7.3	6.9	6.5	5.7	6.3	6.2	6.8	7.0	7.0	59.7
CTL_W_28	7.0	6.7	6.4	5.6	6.0	6.3	6.6	7.0	7.0	58.6
CTL_W_35	6.8	6.6	6.3	5.5	6.0	6.4	6.5	7.0	7.0	58.1
CTL_EA_0	6.0	6.2	6.1	6.0	5.3	5.6	6.2	7.0	7.0	55.4
CTL_EA_7	7.0	6.8	6.3	5.9	6.5	5.7	6.8	7.0	7.0	59.0
CTL_EA_14	8.3	7.4	6.4	5.9	8.0	6.3	7.4	7.0	7.0	63.7
CTL_EA_21	8.6	7.2	6.5	5.8	8.3	6.4	7.5	7.0	7.0	64.3
CTL_EA_28	8.2	7.2	6.6	5.6	8.0	6.5	7.3	7.0	7.0	63.4
CTL_EA_35	8.0	6.8	6.6	5.6	7.5	6.5	7.1	7.0	7.0	62.1

### Major chemical compositions analysis

**Figure 1:**
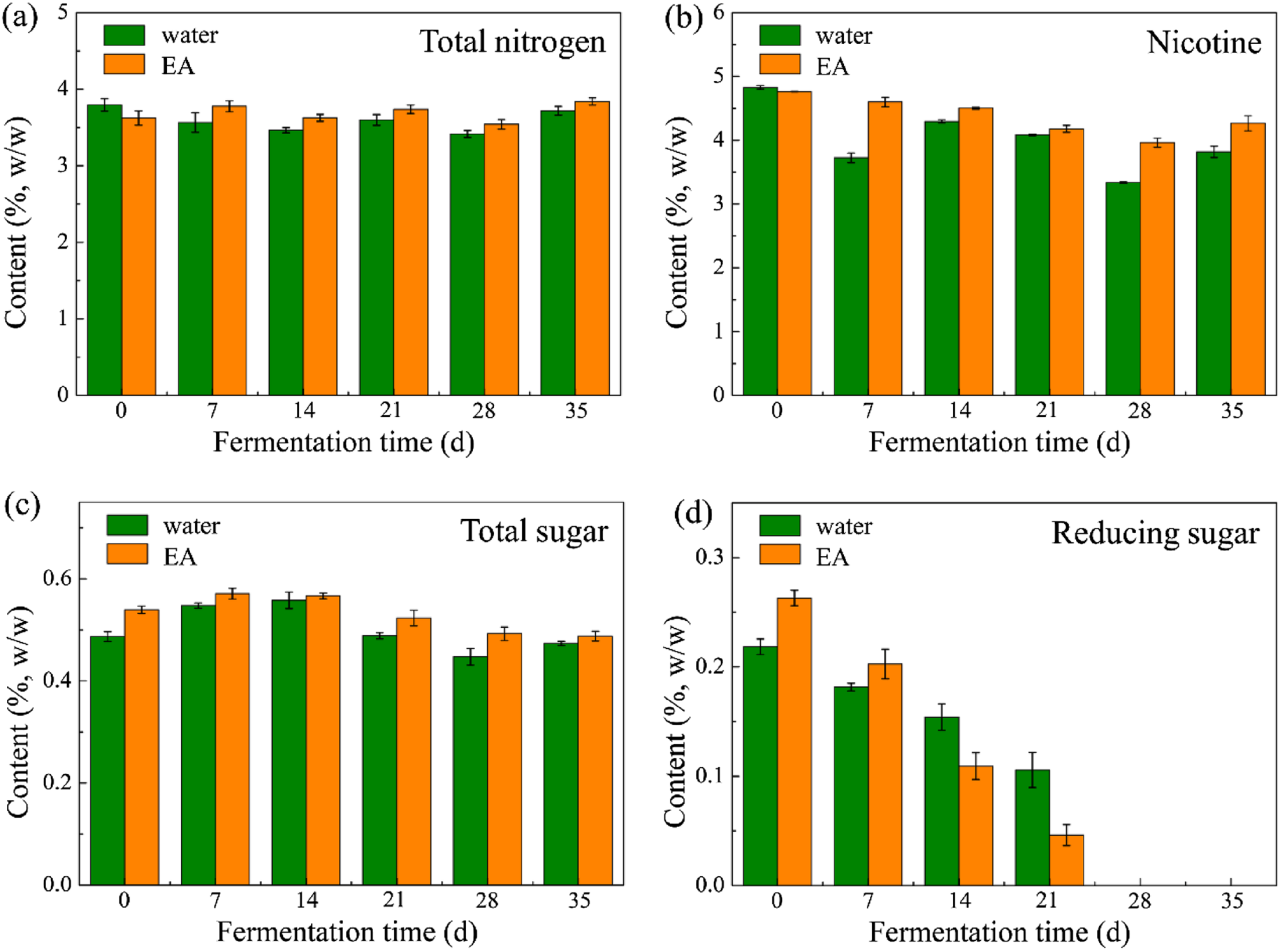
Contents of total nitrogen (**a**), nicotine (**b**), total sugar (**c**), and reducing sugar (**d**) of CTL samples in water- and EA-groups.

Effects of EA on six major chemical components in tobacco leaves, including total nitrogen, nicotine, total sugar, reducing sugar, K, and Cl, were analyzed firstly. The contents of total nitrogen and nicotine were related to the smoke concentration and pungency. As shown in Fig. 1a, the content of total nitrogen in CTL raw material was about 3 ~ 4% (w/w), which is consistent with previous reports^15,21^. Total nitrogen content shows an increased trend with the addition of EA and was affected slightly by the fermentation process. In fact, nicotine is one of the main nitrogenous compounds in CTLs. The changes of nicotine content in DX-4 during fermentation are presented in Fig. 1b. The contents of nicotine were about 4 ~ 5% (w/w) and reduced during the fermentation process, which was due to the degradation of nicotine into nornicotine through nitrosation reaction^22^. Besides, with the introduction of EA, the nicotine contents of CTLs were improved. In the smoking process, nicotine would degrade into nitrogen heterocyclic aromatic components, such as pyrrole, pyridine, and pyrazine compounds, which endows the CTLs with fragrant baked and caramel aromas^23^. Therefore, adding EA in the fermentation process could improve the sensory quality of cigar.

Sugar presents a significant influence on the sensory quality of tobacco, which could weaken the acrimonious taste of the smoke by producing acids and be convert to various aromatic substances by Maillard reaction, caramelization, and pyrolysis reaction^24,25^. Additionally, the content of sugar is related to the sweetness of CTLs. The effects of EA on the contents of total sugar and reducing sugar were shown in Fig. 1c,d. About 0.5% (w/w) of total sugar and 0.2% (w/w) of reducing sugar could be detected in CTL raw materials. Compared with flue-cured tobacco, cigar leave presents a relatively low level on sugar content^21^. As the fermentation proceed, the total sugar content increased firstly and then decreased. The hydrolysis of starch, cellulose, and pectin, as well as the consumption of sugars resulted in the insignificant change of total sugar content. Besides, the addition of EA can increase the total sugar and reducing sugar contents in CTLs. The consumption rate of reducing sugar in EA-group was higher than that in water-group, which was due to the acceleration of biochemical process with the abundance of substrates. At the end of the fermentation process, reducing sugar can hardly be detected, because reducing sugar is almost completely consumed by biochemical reactions, such as Maillard reaction^26^.

**Figure 2:**
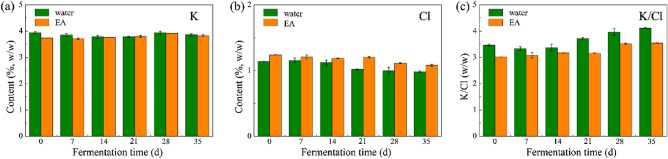
Contents of K (**a**), Cl (**b**), and K/Cl values (**c**) of CTL samples in water- and EA-groups.

It is reported that K and Cl show the significant influence on the combustibility of tobacco leaves^26,27^. The weight ratio of K and Cl (K/Cl) and the content of K present a positive correlation with combustibility. As shown in Fig. 2, 3.7 ~ 4.0% of K could be detected in CTLs, which shows no obvious change in the content during fermentation. By contrast, the contents of Cl decreased during the fermentation process, which may due to the volatilization of chlorinated compounds. As a result, the increased value of K/Cl was found as fermentation proceed. Besides, the Cl content in CTL increased with the introduction of EA, since EA was prepared by tap water as solvent and Cl could be found in solvent. Although the value of K/Cl decreased as adding EA, the relatively high content of K and value of K/Cl were still detected, indicating that the EA-treated CTLs possessed a good performance in combustibility.

### Nonvolatile organic acid analysis

**Figure 3:**
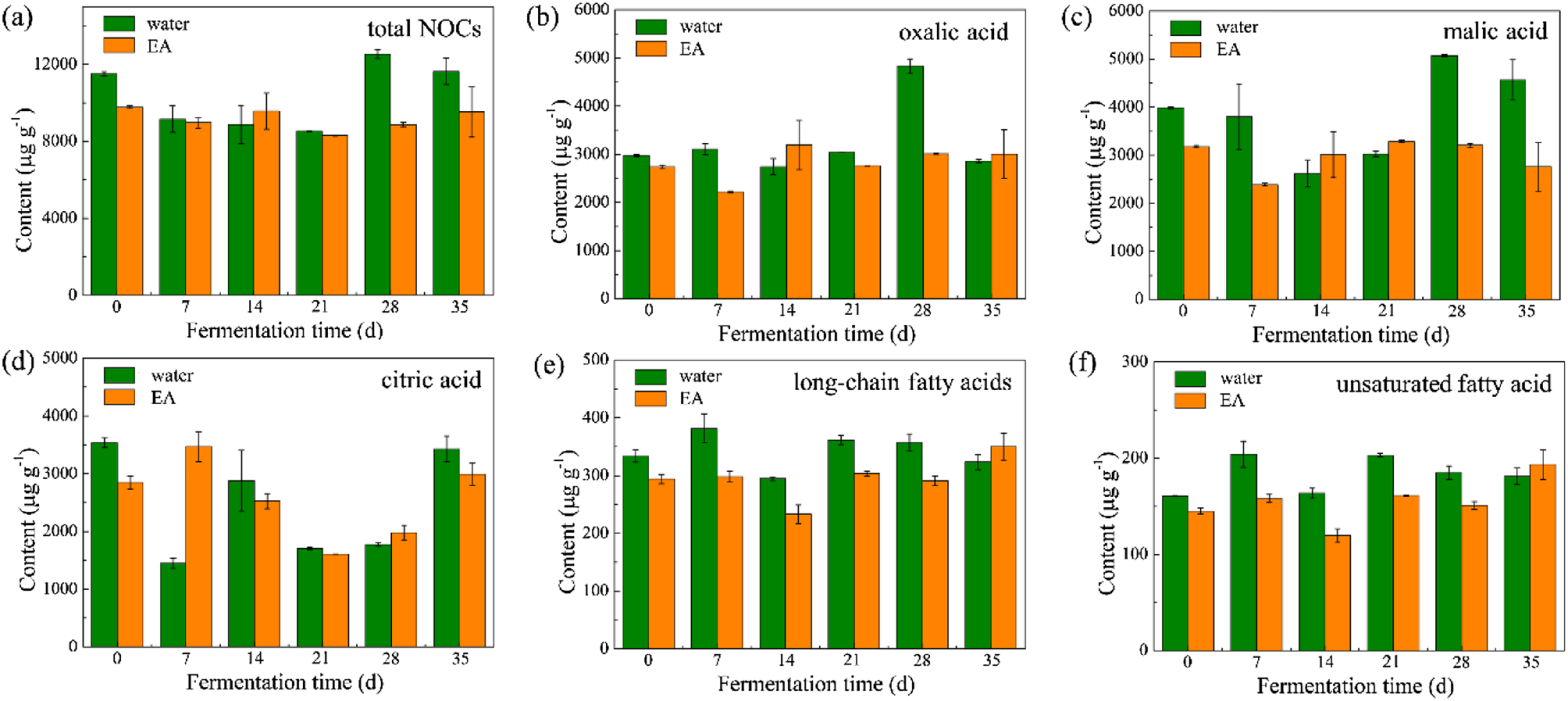
Contents of total NOCs (**a**), oxalic acid (**b**), malic acid (**c**), citric acid (**d**), long-chain fatty acids (**e**), and unsaturated fatty acid (**f**) of CTL samples in water- and EA-groups.

Nonvolatile organic acid (NOC) is an important component in CTLs, which accounts for 70 ~ 80% of organic acid and affects the sensory quality of cigar significantly^27^. NOC plays an important role in changing the pH and fragrance of flue gas, thus regulates the mellowness and irritation of CTLs. In this study, fourteen NOCs were detected, as shown in Table S1. The total content of NOCs was reduced as adding EA, which was resulted by the alcohols from EA (Fig. 3). The consumption of NOCs by EA would weaken the reaction between NOC and NIC, thus the NIC contents increased with the addition of EA, which is consistent with the results of Fig. 1b. The total content of NOCs decreased firstly and then increased, which showed a similar value at the beginning and end of the fermentation process. However, the variation amplitude of NOC content in EA-group was gentler than that in water-group. It means that EA built an environment similar with buffer system, which would be beneficial to maintain the acid–base balance of CTLs and improve the fluentness of cigars. Among the fourteen NOCs, oxalic acid, malic acid, and citric acid accounts for 90% (w/w) of NOCs, which would affect the irritation and smoke concentration obviously. As exhibited in Fig. 3, the contents of these three acids in EA-group were lower than that in water-group at the beginning and end of the fermentation. The change rule of the three acids content of EA-group in 0 ~ 28 days was similar with that of water-group in 7 ~ 35 days, indicating that the addition of EA accelerated the generation and consumption of NOCs. This phenomenon may be related with the changes of microbial activities with EA, which would be discussed combined with microbial community analysis in the following. It was reported that malic acid possessed a positive effect on the quality of tobacco leaves, while oxalic acid and citric acid would damage the taste of tobacco leaves^28^. Accordingly, 21 days may be an optimal fermentation period based on the NOCs analysis for EA-group. Besides, several long-chain fatty acids, including two unsaturated fatty acid (oleic acid and linoleic acid) and four saturated fatty acid (myristic acid, palmitic acid, stearic acid, and arachidic acid), were detected in this study. Unsaturated fatty acid would increase the irritation and reduce the smoothness of smoke, while saturated fatty acid would increase the mellowness of smoke^29^. Therefore, the relatively low content of long-chain fatty acids, especially unsaturated fatty acid in EA-group implies its possibly good performance for sensory.

### Aroma components analysis

Various aroma components were reported to detect in cigar leaves, which plays an important role in the sensory quality of CTLs^30^. Therefore, effects of EA on the contents of aroma components were analyzed by GC–MS and shown as Table 2 and Table S2. As we can see, the total contents of aroma components increased from 1.6738 mg g^−1^ to 1.8076 mg g^−1^ in 35 days as adding EA. In fact, during the fermentation process, the contents of aroma components were improved with the addition of EA. Higher content of aroma components would endow CTLs with more fragrant flavor. In this study, various kinds of aroma components were detected, including alcohols, alkenes, phenolics, ketones, acids, esters, and heterocycles of compounds. As shown in Table 2, the contents of alcohols, alkenes, ketones, acids, and esters were higher in EA-group than that in water-group. Since the sum of alcohols, alkenes, and ketones account for 80 ~ 90% (w/w) of the total aroma components, and ketones contributed mostly to the aroma value of cigar leaves, CTL samples treated with EA would show an improved performance on sensory quality. Additionally, it shows a trend of first increase and then decrease on the contents of aroma components during the fermentation process. In 14 days, a highest value of aroma component content was found. It indicated that the optimal fermentation time may be 14 days.

**Table 2:** Contents of aroma components of CTL samples in water- and EA-groups.

Components	Contents (mg g^−1^)											
	Water						EA					
	0 day	7 days	14 days	21 days	28 days	35 days	0 day	7 days	14 days	21 days	28 days	35 days
**Various kinds of aroma components**												
Alcohols	0.6389	0.7553	0.9445	0.8372	0.6975	0.6151	0.8826	0.8585	1.0839	0.9242	0.7071	0.6459
Alkenes	0.8176	0.9196	0.9850	0.9535	0.6377	0.5213	0.9216	0.9213	1.0336	0.9632	0.6926	0.5737
Phenolics	0.1464	0.1460	0.1156	0.1136	0.0996	0.0865	0.1407	0.1253	0.1146	0.0823	0.0799	0.0787
Ketones	0.3356	0.3849	0.4617	0.3792	0.3165	0.2919	0.4546	0.5415	0.5758	0.3803	0.3254	0.3169
Acids	0.0871	0.0826	0.0877	0.0770	0.0682	0.0741	0.1880	0.0866	0.0921	0.0858	0.0969	0.1073
Esters	0.0794	0.0750	0.0755	0.0807	0.0813	0.0531	0.1441	0.1290	0.1154	0.1097	0.1178	0.0964
Heterocycles	0.1952	0.1923	0.1825	0.1541	0.1251	0.1059	0.1748	0.1890	0.1390	0.1114	0.1113	0.0960
Sum	2.2131	2.4731	2.7648	2.5183	1.9577	1.6738	2.7184	2.7646	3.0623	2.5711	2.0341	1.8076
**Representative aroma components**												
Neophytadiene	0.5429	0.6483	0.7518	0.6979	0.3990	0.3267	0.6057	0.6545	0.7950	0.7454	0.4867	0.4006
Megastigmatrienone I	0.0134	0.0178	0.0149	0.0128	0.0117	0.0105	0.0176	0.0209	0.0184	0.0129	0.0139	0.0174
Megastigmatrienone II	0.0211	0.0227	0.0186	0.0183	0.0170	0.0139	0.0267	0.0305	0.0301	0.0181	0.0183	0.0103
5,9,13-pentadecatrien-2-one, 6,10,14-trimethyl-, (E,E)-	0.0181	0.0196	0.0259	0.0287	0.0239	0.0180	0.0328	0.0445	0.0485	0.0387	0.0324	0.0282
Dihydroactinidiolide	0.0249	0.0262	0.0103	0.0118	0.0106	0.0109	0.0267	0.0287	0.0119	0.0125	0.0134	0.0143
Solanone	0.0149	0.0141	0.0539	0.0293	0.0235	0.0123	0.0111	0.0211	0.0496	0.0259	0.0227	0.0201
3-hydroxy-.beta.-damascone	0.0074	0.0134	0.0076	0.0115	0.0106	0.0109	0.0117	0.0140	0.0117	0.0128	0.0199	0.0092

Except for the total content of aroma components, several representative aroma components were also affected by EA and fermentation process. As shown in Table 2, representative aroma substances such as neophytadiene, megastigmatrienone, and dihydroactinidiolide showed an increasing tread with the addition of EA. As a flavoring substance with the highest content and important intermediate compound of biochemical reaction in tobacco, neophytadiene not only directly affects the sensory quality of tobacco leaves, but also affects the formation and consumption of other flavoring substances^31^. Neophytadiene possesses delicate fragrance, which is formed by the degradation of chlorophyll in the processes of maturation and modulation^32^. As a result, the degradation of chlorophyll with green offensive odor, as well as the generation of neophytadiene, could promote the improvement of mellowness and the reduction of stimulation. At the end of fermentation, the content of neophytadiene increased from 0.3267 mg g^−1^ to 0.4006 mg g^−1^ with the addition of EA (Table 2). Compared with neophytadiene, megastigmatrienone shows a relatively low content in CTLs. However, megastigmatrienone contributes greatly to the aroma quality of cigar due to the low aroma threshold^33^. Megastigmatrienone possesses fruit and tobacco aroma, which usually exists with multiple isomers. In this study, two isomers of megastigmatrienone were detected. The total contents of megastigmatrienone increased from 0.0244 mg g^−1^ to 0.0277 mg g^−1^ in DX-4 as adding EA. The enrichment of megastigmatrienone can effectively improve the mellowness, sweetness, and cleanliness of CTLs^34^. Only light fruit and baked aromas were emanated from dihydroactinidiolide, but it can weaken the pungency well, thus increasing the cleanliness and mellowness of tobacco leaves^35^. Besides of the above-mentioned compounds, the increasement of other aroma components could also lay a material foundation for the quality improvement of cigars (Table S2).

### Microbial community analysis

In order to investigate the effects of EA and fermentation process on the microbial community of CTLs, the distinct regions of 16S rRNA and ITS genes were amplified for Illumina paired-end sequencing. The coverage of all sample was higher than 99.88% for the bacterial and fungal sequences (Table 3), indicating that the sequencing depth of each sample was sufficient to reflect the bacterial and fungal composition of CTL samples. Alpha diversity metrics, including Shannon, Simpson, Chao, and ace, are analyzed to reflect the diversity and richness of the microbial community in samples. The Chao and ace were used to identify the community richness, while Shannon and Simpson were adopted to evaluate community diversity. As shown in Table 3, the richness and diversity of bacterial community in EA-group were lower than that in water-group at the early stage of fermentation, whereas it showed a contrary law at the late stage of fermentation. Moreover, the influences of EA on the richness (and diversity) of fungi and bacteria were different. It means that EA could accelerate the growth of fungi in the early stage and provide suitable metabolic environment for bacteria in the late stage of fermentation. For EA-group, the richness of bacterial and fungal community was increased and then decreased with prolonging fermentation time.

**Table 3:** Alpha diversity indexes of microbial communities of CTL samples in water- and EA-groups.

Fermentation time (days)	Shannon index		Simpson index		Ace index		Chao index		Coverage	
	Water	EA	Water	EA	Water	EA	Water	EA	Water	EA
**Bacteria**										
0	0.633	0.127	0.740	0.967	286.412	135.752	215.250	99.000	0.9988	0.9993
7	1.263	0.197	0.470	0.942	201.446	164.776	158.526	120.056	0.9991	0.9992
14	1.235	0.508	0.475	0.782	252.402	159.348	212.500	157.500	0.9992	0.9991
21	1.076	0.515	0.438	0.816	216.047	239.980	169.136	204.333	0.9989	0.9988
28	0.995	1.088	0.484	0.515	269.316	290.214	229.000	271.367	0.9988	0.9989
35	1.352	1.602	0.385	0.303	224.410	153.496	170.000	143.000	0.9992	0.9995
**Fungi**										
0	1.429	1.632	0.352	0.349	126.898	139.521	96.000	133.000	0.9993	0.9995
7	1.262	2.172	0.446	0.183	149.649	166.172	108.000	169.429	0.9993	0.9996
14	1.206	2.280	0.508	0.194	202.201	173.692	158.313	174.500	0.9993	0.9999
21	1.062	1.877	0.501	0.213	126.394	169.451	88.250	159.615	0.9996	0.9994
28	1.750	1.392	0.311	0.392	206.835	78.489	157.053	58.667	0.9992	0.9997
35	2.118	1.507	0.191	0.442	164.413	172.663	149.440	127.250	0.9993	0.9992

**Figure 4:**
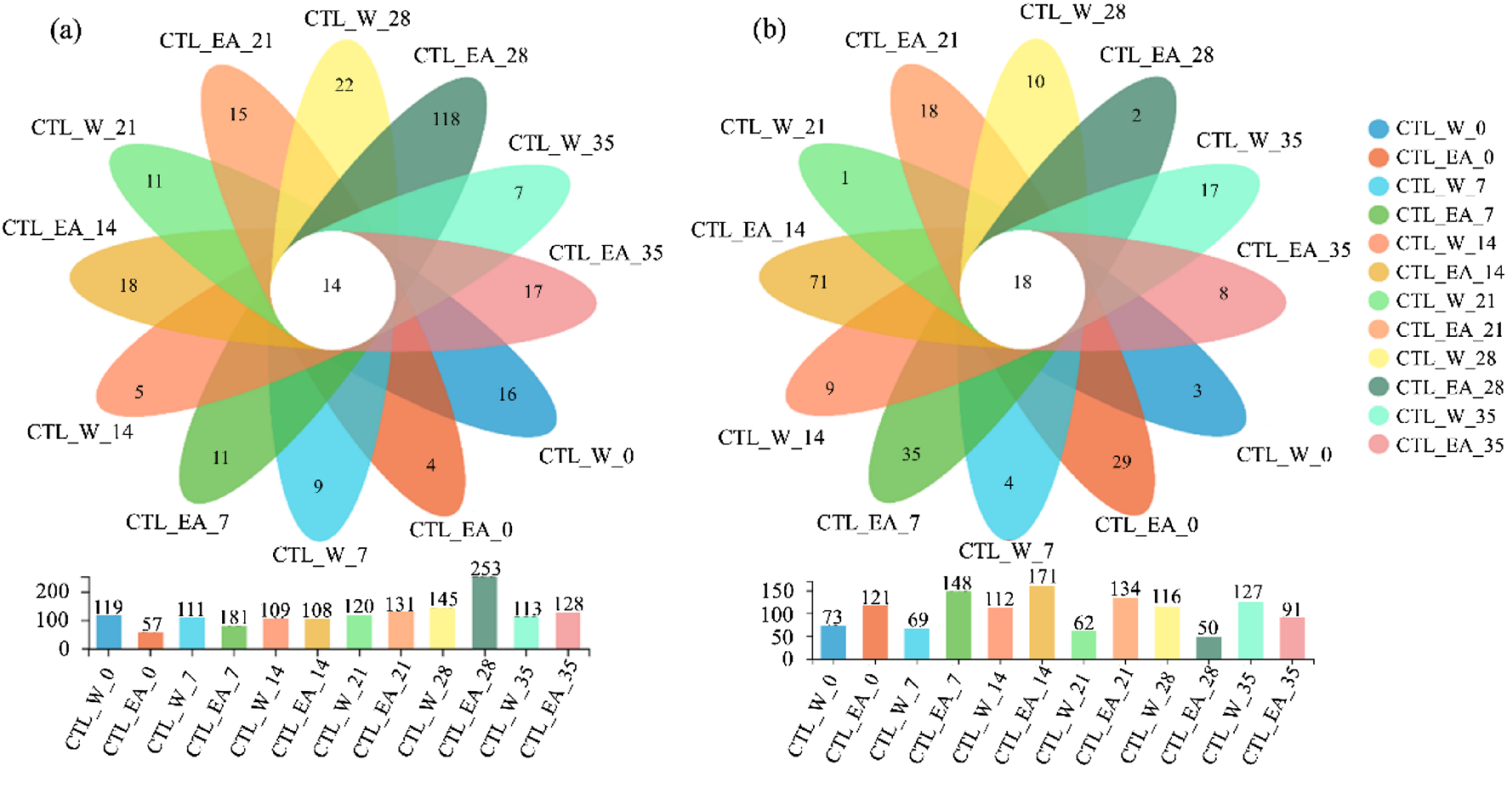
Venn diagram of OTU distribution of bacterial (**a**) and fungal (**b**) communities in different CTL samples.

As illustrated in Fig. 4), 57 ~ 253 bacterial OTUs and 50 ~ 171 fungal OTUs with 97% similarity were found in CTL samples. It showed a low proportion of common OTUs in total OTUs, which means that the unique functional flora in the fermentation process showed an obvious succession phenomenon. The quantity of fungal unique OTUs in EA-group was higher than that in water-group at 0 ~ 21 days, indicating that EA could promote the growth of peculiar fungal colonies in CTLs. Besides, the unique bacterial OTUs in CTL_EA_28 was 118, accounting for 46.64% of the total OTUs, indicating that 28 days of fermentation time is beneficial to the bacterial diversity of tobacco leaves. According to the analysis of alpha-diversity, it could be concluded that excessive fermentation is not conducive to the growth and metabolism of microorganisms in CTLs.

**Figure 5:**
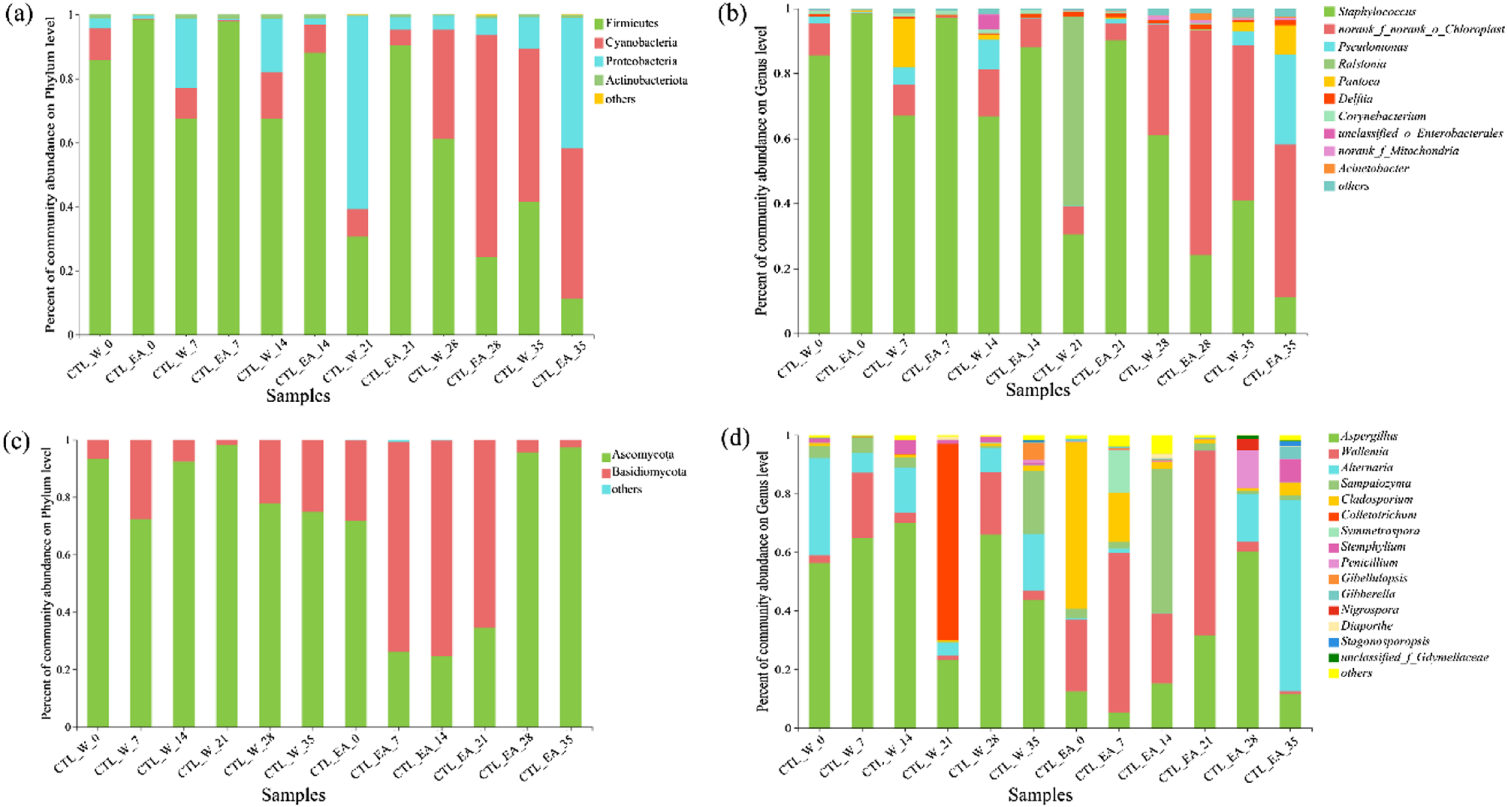
Relative abundance of bacterial community on phylum level (**a**) and species level (**b**) in different CTL samples; relative abundance of fungal community on phylum level (**c**) and species level (**d**) in different CTL samples.

Figure 5a showed the changes of bacterial communities on phylum level during the fermentation. Four different phyla, including *Firmicutes*, *Cyanobacteria*, *Proteobacteria*, and *Actinobacteriota*, were identified among these CTL samples. In the water-group, the relative abundance of *Firmicutes* decreased with prolonging fermentation time, whereas that of *Cyanobacteria* increased. After fermentation, the relative abundances of *Firmicutes* and *Cyanobacteria* were similar. As for the EA-group, *Firmicutes* (over 90%) was the dominant phylum in 0 ~ 21 days, which was more prominent than water-group. However, after 28 days of fermentation, the relative abundance of *Firmicutes* was reduced. At 35 days, *Cyanobacteria* and *Proteobacteria* were the dominant phyla in EA-group. The effects of EA and fermentation on bacterial community at species level were also analyzed (Fig. 5b). Among these species, *Staphylococcus*, *norank_f_norank_o_choroplast*, *Pseudomonas*, and *Ralstonia* were the main dominant bacterial species in CTLs. In the early stage of fermentation, *Staphylococcus* possessed an obvious preponderance with the abundance over 70%, which was consistent with other reports^13,36^. It is observed that the addition of EA further enhanced the dominance of *Staphylococcus*. Sugar was one of the main components in EA, which could provide metabolic substrates for the growth of *Staphylococcus* and promote the abundance of *Staphylococcus*. Accordingly, the generation of metabolite, i.e. acids, produced by *Staphylococcus* was accelerated. In the late stage of fermentation, the relative abundances of *Staphylococcus* decreased due to the consumption of reducing sugar (Fig. 1d). As a result, *norank_f_norank_o_choroplast* and *Pseudomonas* were the most frequently detected species in CTL samples.

In addition to bacteria, relative abundance of fungal community was also analyzed. As revealed in Fig. 5c, *Ascomycota* with the abundance over 70% was the principal phylum among the six samples in water-group. Among the EA-group, the decreased relative abundance of *Ascomycota* was observed in 7 ~ 21 days, since *Basidiomycota* became the prevalent fungal. In 21 ~ 35 days, *Ascomycota* regained its position as the dominant fungal. As illustrated in Fig. 5d, six different species, including *Aspergillus*, *Wallemia*, *Alternaria*, *Sampaiozyma*, *Cladosporium*, and *Colletotrichum*, were mainly found in CTLs. *Aspergillus* is the dominant species in the fungal community in water-group^37^. It is observed that the relative abundance of *Aspergillus* increased first and then reduced with the extension of fermentation time. Notably, the introduction of EA changed the fungal communities in CTLs significantly. The abundance of *Aspergillus* in EA-group was lower than that in water-group. Since *Aspergillus* poses a relatively high mildew-causing risk for CTLs, the cigar products fermented with EA would possess a reliable health for people. Besides, *Wallemia* was the prevalent fungal in EA-group during 0 ~ 21 days. *Wallemia* could promote the production of flavonoid which would be decomposed into aroma components in the combustion of cigars^38^. Overall, these results illustrated that the addition of EA and fermentation process influenced the relative abundances of bacterial and fungal communities in CTLs obviously. The changes of microbial communities with EA could promote the quality improvement of CTLs.

### Mechanism of the changes in CTLs compositions with EA

**Figure 6:**
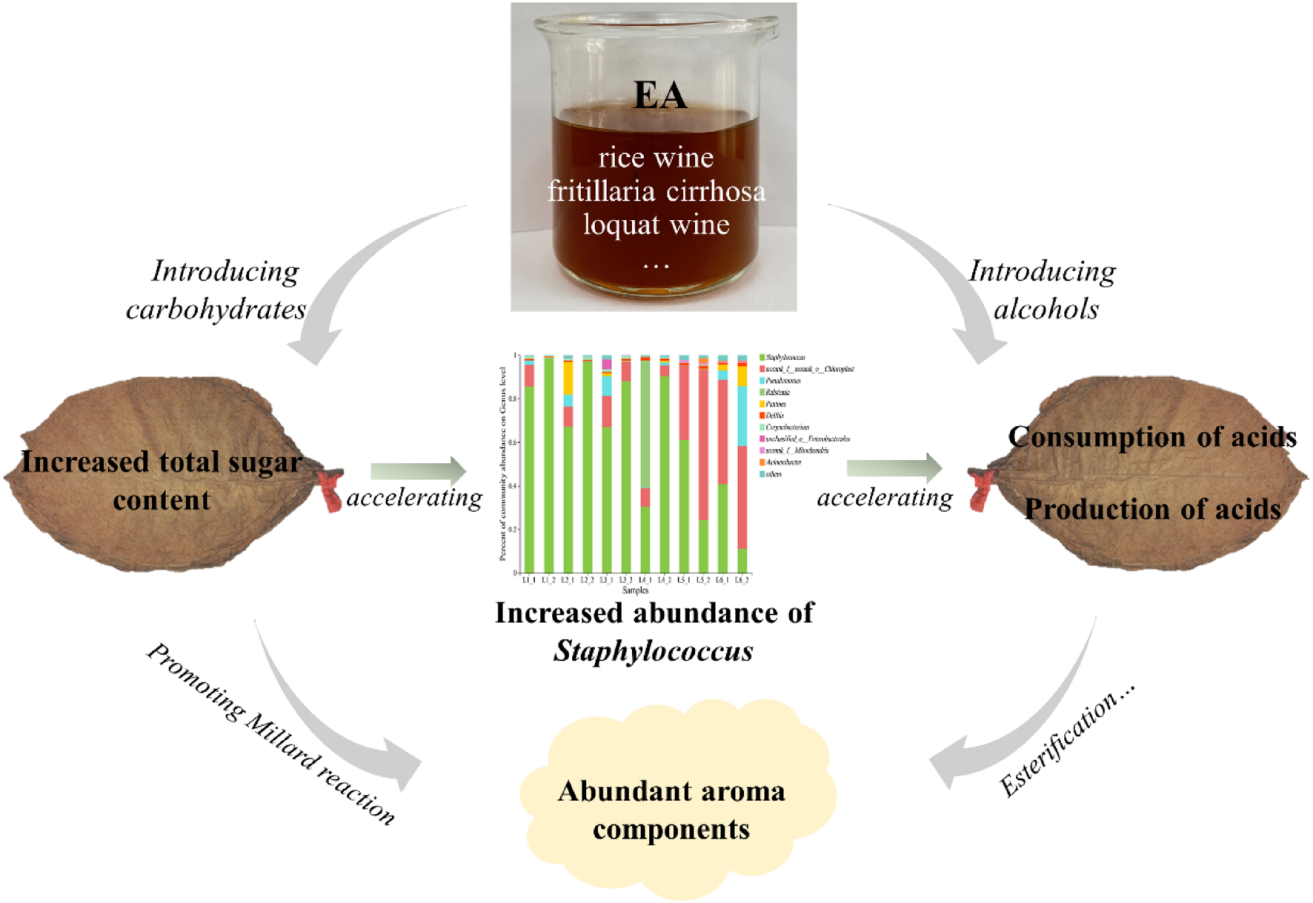
Schematic illustration of mechanism of EA improving the CTLs quality.

In this study, the fermentation of CTLs in EA-group shows several main differences with that in water-group. Firstly, the total contents of total sugar and aroma components increased. Secondly, the consumption rate of reducing sugar was improved, and the generation as well as consumption of NOCs was accelerated. Thirdly, the relative abundances of *Staphylococcus* and *Wallemia* were enhanced. Accordingly, a possible mechanism of EA improving the CTLs quality was proposed, as shown in Fig. 6. The carbohydrates originated from EA could improve the content of total sugar and reducing sugar in CTLs, thus promoting the enrichment of *Staphylococcus* in tobacco leaves. The increased abundance of *Staphylococcus* accelerated the consumption of reducing sugar and production of NOCs. Meanwhile, alcohols from EA were introduced into CTLs, which would promote the formation of esters. Moreover, the relatively high content of total sugar facilitates the proceeding of Maillard reaction. As a result, the contents of aroma components were improved. Overall, the increased contents of total sugar and aroma components would endow the cigar with an improved sensory quality.

## Conclusions

This study investigated the changes of sensory qualities, major chemical compositions as well as microbial communities of CTLs during fermentation with EA. Systematic studies indicated that the addition of EA could increase the contents of total nitrogen, nicotine, total sugar, and aroma components in CTLs, and accelerate the generation and consumption of NOCs. The increased abundance of *Staphylococcus* and decreased abundance of *Aspergillus* were detected. Furthermore, a preliminary exploration about mechanism of the changes in CTLs compositions with EA was conducted. The present study suggested that carbohydrates and alcohols originated from EA promote the enrichment of *Staphylococcus* and accelerate biochemical reactions, thus improving the contents of aroma components. As a results, the aroma quality and concentration, cleanliness, as well as aftertaste of cigar were improved. Therefore, it is believed that EA rich in carbohydrates and aroma components is effective in improving the quality of tobacco leaves. However, more detailed studies of the changes in activities of relevant enzymes during the fermentation of CTLs are required to determine the precise mechanisms of improved CTLs quality with EA. In conclusion, this study not only demonstrates the reasons of flavor enhancement and quality improvement by industrial EA, but also provides strategy to prepare novel EAs.

## Supplementary Information

Supplementary Tables.

## Data Availability

The datasets used and/or analysed during the current study available from the corresponding author on reasonable request.
