# Supplementary material for: Study on the chemical compositions and microbial communities of cigar tobacco leaves fermented with exogenous additive
Source: Sci Rep. 2022 Nov 10;12:19182. doi: 10.1038/s41598-022-23419-y (PMC9649726; doi:10.1038/s41598-022-23419-y)
Supplement: Supplementary file 1 — Supplementary Tables. [file 41598_2022_23419_MOESM1_ESM.pdf]

Supporting information for:

Study on the chemical compositions and microbial communities of cigar tobacco leaves fermented with exogenous additive

Wanrong Hu[1], Wen Cai[1], Zhaojun Zheng[2], Yuanfa Liu[2], Cheng Luo[1], Fang Xue[1], Dongliang Li [1]*

1: *Key Laboratory of Chinese Cigar Fermentation, Center of Technology Innovation for Cigar, China Tobacco Sichuan Industrial Co., Ltd., Chengdu 610000, China*

2: *School of Food Science and Technology, Jiangnan University, Wuxi 214112, China*

Corresponding author:

Dongliang Li

Key Laboratory of Chinese Cigar Fermentation, Center of Technology Innovation for Cigar, China Tobacco Sichuan Industrial Co., Ltd., Chengdu 610000, China

Tel.: 028-86005602; Fax: 028-86005602

Email: 360188228@qq.com (D. Li)

List of Contents

Table S1 Effect of EA on the contents of NOCs in CTL samples.

Table S2 Effect of EA on various aroma components of CTL samples.

Table S1 Effect of EA on the contents of NOCs in CTL samples.

Components	Content (mg g^{-1})											
	Water						EA					
	0 d	7 d	14 d	21 d	28 d	35 d	0 d	7 d	14 d	21 d	28 d	35 d
Lactic acid	13.91±0.08	19.64±0.34	18.93±1.50	16.77±0.10	22.38±1.18	18.86±1.09	19.24±6.50	20.01±0.78	19.25±2.35	15.28±0.04	16.83±0.11	20.03±3.70
Oxalic acid	2971.12±22.67	3106.72±108.07	2739.83±170.35	3042.16±3.56	4833.97±146.75	2858.31±38.29	2739.55±29.62	2216.47±23.32	3191.53±507.85	2758.64±2.63	3010.48±6.12	3003.39±509.21
Malonic acid	250.08±3.03	185.20±6.58	194.44±11.65	197.06±2.87	272.64±12.39	235.32±9.93	263.69±2.44	293.59±0.35	251.14±39.94	174.47±0.18	176.41±0.07	261.41±44.89
Levulinic acid	8.90±0.59	30.33±1.11	9.77±1.29	24.18±0.61	35.17±0.66	13.23±0.80	17.70±2.37	20.24±0.30	15.12±1.43	19.42±0.96	19.34±0.75	19.72±4.59
Succinic acid	397.45±1.14	145.59±5.11	109.11±2.02	144.49±0.45	148.15±3.61	148.26±2.42	409.59±2.82	234.69±1.12	311.47±49.49	107.96±0.43	149.12±0.10	96.84±16.41
Malic acid	3988.02±21.72	3803.59±681.34	2618.61±281.61	3025.34±59.97	5071.19±22.74	4566.43±422.01	3178.57±22.54	2389.10±25.22	3010.54±476.03	3285.41±25.67	3206.35±38.89	2760.10±506.94
Citric acid	3537.63±89.49	1450.65±88.76	2879.13±526.54	1703.80±26.78	1774.34±36.36	3428.98±223.16	2849.87±108.74	3470.29±258.96	2526.23±133.46	1610.17±0.64	1979.88±124.27	2989.58±194.23
Vanillic acid	7.73±0.99	25.15±3.38	13.88±1.12	18.48±2.18	19.16±5.41	31.15±1.86	3.74±0.85	25.61±0.29	16.81±1.17	20.18±0.99	16.47±3.93	28.91±2.78
Myristic acid	16.88±1.25	14.42±1.58	11.34±1.65	11.97±0.25	17.10±0.87	17.01±0.69	15.47±1.33	11.88±0.95	11.64±1.38	13.43±0.02	11.27±1.04	19.47±1.51
Palmitic acid	81.51±1.63	103.20±6.46	71.53±1.26	89.03±1.75	99.58±3.26	80.73±2.98	68.06±1.77	76.13±3.51	61.51±3.67	79.03±0.53	79.53±3.42	91.77±5.20
Linoleic acid	43.30±1.37	63.63±3.54	50.33±2.20	70.87±0.41	70.36±2.71	61.72±2.66	46.97±1.04	53.19±1.18	38.72±3.26	53.62±0.17	49.18±1.28	76.79±5.92
Oleic acid	117.35±1.92	140.68±10.07	113.40±2.88	132.21±1.39	114.48±4.43	119.61±5.81	97.91±2.00	105.10±3.14	80.89±3.57	107.48±0.64	101.79±2.71	116.54±9.26
Stearic acid	60.16±11.30	51.97±2.04	43.63±9.40	50.35±4.40	46.34±2.20	38.31±0.60	42.48±1.34	44.92±0.55	34.71±3.77	44.42±2.54	42.84±0.19	38.65±0.93
Arachidic acid	14.50±1.68	7.59±0.77	4.59±1.97	7.33±0.24	9.40±1.52	6.38±0.47	23.14±0.65	7.20±0.59	5.64±0.31	5.66±0.20	6.58±0.37	7.16±0.59

Table S2. Effect of EA on various aroma components of CTL samples.

	Components	Content (mg g^{-1})											
		Water						EA					
		0 d	7 d	14 d	21 d	28 d	35 d	0 d	7 d	14 d	21 d	28 d	35 d
alcohols	Furan, tetrahydro-2,2,5,5-tetramethyl-	0.0081	0.0081	/	0.0066	0.0045	0.0074	0.0032	0.0022	0.0081	0.0111	/	0.0025
	2-methyl-1-decanol	0.0663	0.0539	0.0543	0.0674	0.0225	0.0500	0.0737	0.0313	0.0154	0.0362	0.0232	0.0369
	7-Oxabicyclo[4.1.0]heptan-3-ol, 6-(3-hydroxy-1-butenyl)-1,5,5-trimethyl-	0.0465	0.0672	0.038	0.0614	0.0246	0.0523	0.0236	0.0642	0.0525	0.0509	0.0342	0.0306
	Cedran-diol, (8S,14)-	0.0210	0.0369	0.0699	0.0665	0.0531	0.0360	0.0665	0.0898	0.0778	0.0821	0.0635	0.0608
	(+)-(S)-dehydrovomifoliol	0.0548	0.0689	0.0649	0.0780	0.0649	0.0743	0.0662	0.0745	0.0866	0.0705	0.0445	0.0471
	4,8,13-Cyclotetradecatriene-1,3-diol, 1,5,9-trimethyl-12-(1-methylethyl)-	0.0169	0.0262	0.0541	0.0312	0.0103	0.0191	0.0323	0.0412	0.0388	0.0713	0.0205	0.0102
	Thunbergol	0.0376	0.0296	0.0585	0.0423	0.0159	0.0311	0.0573	0.0474	0.0677	0.0511	0.0640	0.0306
	Phytol	0.1774	0.2289	0.3525	0.3256	0.2687	0.1412	0.2234	0.2429	0.4551	0.3655	0.2693	0.2040
	trans-Geranylgeraniol	0.0103	0.0189	0.0182	0.0174	0.0161	0.0109	0.0325	0.0274	0.0241	0.0169	0.0108	0.0203
	Cholesterol	0.0279	0.036	0.0359	0.0208	0.0582	0.0377	0.0626	0.0381	0.0401	0.0222	0.0433	0.0309

	Campesterol	0.0389	0.0448	0.0573	0.0272	0.0288	0.0289	0.0859	0.0561	0.0526	0.0567	0.0318	0.0451
	Stigmasterol	0.1206	0.1181	0.1189	0.0821	0.0941	0.1021	0.1291	0.1236	0.1396	0.0711	0.0684	0.1074
	.gamma.-Sitosterol	0.0126	0.0178	0.022	0.0107	0.0358	0.0241	0.0263	0.0198	0.0255	0.0186	0.0336	0.0195
	2,4-Dimethyl-1-heptene	0.0436	0.0547	0.0175	0.0215	0.0478	0.0133	0.0183	0.0382	0.0109	0.0394	0.0473	0.0125
	4-Decene, 7-methyl-, (E)-	0.0063	0.0071	0.0026	0.0055	0.0026	0.0038	0.0076	0.0081	0.0081	0.0035	0.0030	0.0033
	(+)-Dipentene	0.0069	0.008	0.0077	0.0054	0.0089	/	0.0112	0.0051	0.0067	0.0033	0.0036	/
	1-Decene, 2,4-dimethyl-	0.0644	0.0654	0.0506	0.0454	0.0326	0.0379	0.0737	0.0547	0.0510	0.0386	0.0284	0.0257
	7-methyl-undecene	0.0403	0.0409	0.0202	0.0394	0.0301	0.0301	0.0507	0.0378	0.0144	0.0194	0.0337	0.0164
alkenes	2-Decene, 7-methyl-, (Z)-	0.0169	0.0126	0.0199	0.0486	0.0226	0.0368	0.0202	0.0244	0.0163	0.0561	0.0241	0.0493
	2,4,6,8-Tetramethyl-1-undecene	0.0058	/	0.0166	0.0077	0.0068	0.0068	0.0059	0.0042	0.0067	0.0099	/	/
	.beta.-Guaiene	0.0139	0.0128	0.0087	0.0109	0.0091	0.0038	0.0117	0.0146	0.0164	0.0086	0.0025	0.0078
	Neophytadiene	0.5429	0.6483	0.7518	0.6979	0.399	0.3267	0.6057	0.6545	0.7950	0.7454	0.4867	0.4006
	Bicyclo[3.1.1]hept-2-ene, 2,2'-(1,2-ethanediyl)bis[6,6-dimethyl-	0.0766	0.0698	0.0894	0.0712	0.0782	0.0621	0.1166	0.0797	0.1081	0.0390	0.0633	0.0581
	2,4-Di-tert-butylphenol	0.1188	0.1228	0.0867	0.0746	0.0663	0.0562	0.1063	0.0911	0.0803	0.0564	0.0451	0.0443
Phenolics	Phenol, 2,2'-methylenebis[6-(1,1-	0.0075	0.0050	0.0069	0.0096	0.0081	0.0105	0.0071	0.0099	0.0085	0.0051	0.0083	0.0035

	dimethylethyl)-4-methyl-												
	Vitamin E	0.0201	0.0182	0.0220	0.0294	0.0252	0.0198	0.0273	0.0243	0.0258	0.0208	0.0265	0.0309
	Solanone	0.0149	0.0141	0.0539	0.0293	0.0235	0.0123	0.0111	0.0211	0.0496	0.0259	0.0227	0.0201
	1-Nonene, 4,6,8-trimethyl-	0.0267	0.0255	0.0145	0.0232	0.0108	0.0181	0.0114	0.0136	0.0201	0.0152	0.0119	0.0107
ketones	Ethanone, 1-(1a,2,3,5,6a,6b-hexahydro-3,3,6a-trimethyloxireno[g]benzofuran-5-yl)-	0.0079	0.0067	0.0050	0.0056	0.0051	0.0057	0.0107	0.0094	0.0092	0.0086	0.0077	0.0041
	Megastigmatrienone I	0.0134	0.0178	0.0149	0.0128	0.0117	0.0105	0.0176	0.0209	0.0184	0.0129	0.0139	0.0174
	3-Hydroxy-.beta.-damascone	0.0074	0.0134	0.0076	0.0115	0.0106	0.0109	0.0117	0.0140	0.0117	0.0128	0.0199	0.0092
	Megastigmatrienone II	0.0211	0.0227	0.0186	0.0183	0.017	0.0139	0.0267	0.0305	0.0301	0.0181	0.0183	0.0103
	2-Cyclohexen-1-one, 4-(3-hydroxy-1-butenyl)-3,5,5-trimethyl-	0.0081	0.0293	0.0186	0.0206	0.0159	0.0133	0.0219	0.0243	0.0228	0.0138	0.0104	0.0106
	6-Hydroxy-4,4,7a-trimethyl-5,6,7,7a-tetrahydrobenzofuran-2(4H)-one	0.0455	0.0510	0.0323	0.0441	0.0385	0.0337	0.0376	0.0463	0.0504	0.0486	0.0274	0.0321
	3-Ethyl-3,4-dihydro-2(1H)-quinoxalinone	0.1005	0.0903	0.1511	0.0830	0.0858	0.0946	0.1941	0.1704	0.1771	0.0946	0.0904	0.0889

	2-Pentadecanone, 6,10,14-trimethyl-	0.0353	0.04	0.063	0.0544	0.0463	0.0237	0.0439	0.0629	0.0786	0.0458	0.0441	0.0407
	5,9,13-Pentadecatrien-2-one, 6,10,14-trimethyl-, (E,E)-	0.0181	0.0196	0.0259	0.0287	0.0239	0.0180	0.0328	0.0445	0.0485	0.0387	0.0324	0.0282
	7,11-Epoxymegastigma-5(6)-en-9-one	0.0292	0.0401	0.0493	0.0421	0.0225	0.0267	0.0280	0.0706	0.0504	0.0369	0.0205	0.0375
	2-Buten-1-one, 1-(2,6,6-trimethyl-1-cyclohexen-1-yl)-	0.0075	0.0144	0.007	0.0056	0.0049	0.0105	0.0071	0.0130	0.0089	0.0084	0.0058	0.0071
Acids	Pentanoic acid, 3-methyl-	0.0192	0.0274	0.0146	0.0114	0.015	0.0206	0.0236	0.0211	0.0184	0.0111	0.0342	0.0145
	n-Hexadecanoic acid	0.0536	0.0398	0.0502	0.0421	0.0373	0.0363	0.1449	0.0481	0.0540	0.0498	0.0452	0.0644
	Octadecanoic acid	0.0143	0.0154	0.0229	0.0235	0.0159	0.0172	0.0195	0.0174	0.0197	0.0249	0.0175	0.0284
Esters	Dihydroactinidiolide	0.0249	0.0262	0.0103	0.0118	0.0106	0.0109	0.0267	0.0287	0.0119	0.0125	0.0134	0.0143
	4,7,10,13,16,19-Docosahexaenoic acid, methyl ester, (all-Z)-	0.0207	0.0201	0.0219	0.0233	0.0164	0.0169	0.0408	0.0301	0.0298	0.0204	0.0205	0.0191
	Sclareolide	0.0338	0.0287	0.0433	0.0456	0.0543	0.0253	0.0766	0.0702	0.0737	0.0768	0.0839	0.0630
heterocycles	Ethosuximide	0.0141	0.0078	0.0052	0.0062	0.0051	0.0058	0.0115	0.0106	0.0117	0.0060	0.0068	0.0045
	2,5-Dimethyl-1H-indol-6-amine	0.0682	0.0455	0.0474	0.0327	0.0157	0.0152	0.0569	0.0369	0.0233	0.0209	0.0168	0.0147
	Pyridine, 3,3'-(2,4-piperidinediyl)bis-	0.0285	0.0472	0.0290	0.0256	0.0264	0.0157	0.0302	0.0431	0.0239	0.0208	0.0177	0.0109

4-(4-Methyl-piperidin-1-yl)-phenylamine	0.0571	0.0787	0.0778	0.0694	0.0610	0.0563	0.0555	0.0819	0.0685	0.0514	0.0522	0.0507
.beta.-Amyrin	0.0273	0.0131	0.0231	0.0202	0.0169	0.0129	0.0207	0.0165	0.0116	0.0123	0.0178	0.0152

"/" represents the compound was not detected.
